# Safety reporting in neonatal clinical trials: reflections towards optimal, globally relevant approaches

**DOI:** 10.1186/s13063-025-08723-y

**Published:** 2025-02-09

**Authors:** Louise F. Hill, Christina W. Obiero, Adrie Bekker, Ann Sarah Walker, Julia A. Bielicki, Mike Sharland, Francesca Schiavone

**Affiliations:** 1 Centre for Neonatal and Paediatric Infection, University of London, City St George’s, London, UK; 2 Clinical Research Department, KEMRI-Wellcome Trust Research Programme, Kilifi, Kenya; 3 Department of Pediatric and Child Health, Stellenbosch University, Cape Town, South Africa; 4 MRC Clinical Trials Unit at University College London, London, UK; 5 Centre for Tropical Medicine and Global Health, Nuffield Department of Medicine, University of Oxford, Oxford, UK; 6 Paediatric Research Centre, University of Basel Children’s Hospital, Basel, Switzerland

**Keywords:** Adverse events, Safety, Neonates, Clinical trials

## Abstract

Adverse event (AE) collection is a key part of evidence generation in clinical trials and an integral element of safety reporting. AE assessment and documentation is particularly challenging in neonates who are a heterogeneous population with high rates of co-morbidities. Neonatal research is finally gaining the attention of regulators regarding drug development and the need for optimal dosing specific to this population. However, further efforts are necessary to ensure that adverse events (AEs) are adequately collected, allowing for the generation of essential safety data. It is also crucial that the methodology used aligns with the intended trial outcomes to minimise the burden on trial sites. In resource-constrained settings, where pharmacovigilance implementation can be particularly challenging, a pragmatic approach to safety reporting is even more important given the significant public health need for effective drugs. This commentary reflects on some of the challenges and potential areas of improvement in safety reporting that could be addressed in future neonatal-focused trials.

**Supplementary Information:**

The online version contains supplementary material available at 10.1186/s13063-025-08723-y.

## Commentary

The primary aims of randomised controlled trials (RCTs) of investigational medicinal products (IMP) are to establish the efficacy or effectiveness of new or existing drugs whilst also informing safety assessments to evaluate the overall risk–benefit profile of the treatment. The assessment of drug safety in trials is achieved, in part, through the systematic collection and monitoring of adverse events (AEs), as per recognised international guidance [1]. Safety data derived from RCTs or pharmacokinetic (PK) studies in neonates are lacking due to a reluctance to conduct research in this population. This hesitation stems from the complexities involved in undertaking neonatal trials and the absence of international guidance for the assessment and collection of safety data in neonates [2, 3]. This commentary aims to address key issues associated with safety reporting in neonatal-focussed RCTs. It highlights the challenges of conducting clinical trials in this important population and suggests ways of tailoring trials to this context and addressing these complexities.

## Neonatal-focused randomised controlled trials

Neonatal RCTs are challenging to conduct for many reasons. Financial, methodological and ethical considerations contribute to a reluctance in industry to conduct RCTs [4, 5]. Therefore, research in neonatal settings tends to be led from universities, academic networks and more generally Clinical Trials Units (CTUs, specialised biomedical research facilities, that design, undertake and publicise RCTs). The academic research community has progressively attempted to harmonise research methods for the design and conduct of clinical trials in neonates, but difficulties remain on how to both promote and share good practice and ensure data and results generated from neonatal clinical trials are robust and generalisable [6, 7].

The challenges of RCTs in this population are multiple and include the following: high morbidity and mortality impacting survival rates as well as the burden of safety reporting [5]; the heterogenous nature of the neonatal population where broad ranging gestational and postnatal ages influence physiological maturation and can lead to wide variations in PK and pharmacodynamics [8]; prolonged follow-up with financial implications, which may be required to ascertain the longer term effects of drug exposure; as well as practical difficulties such as obtaining consent when parents/guardians are not resident in the admitting hospital or barriers to collecting blood samples due to difficult venous access or small total blood volumes in very low birthweight neonates [9]. This disinclination to conduct trials in neonates means safety data in this vulnerable and unique population are limited [3].

The use of off-label and guideline-divergent medications in neonatal clinical settings [10–12] remains high leading to concerns around safety in terms of over and underdosing as well as the risk of side effects. Off-label use often proceeds due to clinical need prior to RCT data availability [6]. Many stakeholders, including clinicians, trialists, regulators and parents, advocate for improved drug development and enhanced regulatory processes to ensure safe treatments are available to neonates [5]. Increasingly, national and international regulatory frameworks have been put in place to provide incentives for pharmaceutical companies to develop medicines in neonates. New legislation was introduced in the European Union and the United States of America (U.S.A.) in 2007 and 2009 respectively, to encourage pharmaceutical companies to explore the extension of labels to paediatric populations, including neonates [13]. Incentives include 10 years of market protection if a paediatric-use marketing authorisation is granted through the European Medicines Agency [14] and the Best Pharmaceuticals for Children Act grants 6 months of additional market exclusivity for drugs studied in Food and Drug Administration-requested paediatric trials [15]. Other organisations, such as the World Health Organization (WHO), have made significant efforts to encourage the pre-clinical and clinical development of antibiotics, for example, to address conditions such as neonatal sepsis, which have high associated morbidity and mortality particularly in resource-constrained settings [16]. This advocacy is particularly important given the limited RCT data supporting the licencing of antibiotics in neonates. Despite these incentives and recommendations, both the development of new treatments and the repurposing of existing ones for the neonatal population remain inadequate, impacting the generation of neonatal safety data for vital medications.

## Pharmacovigilance activities in neonatal research: the challenges

Pharmacovigilance requirements have evolved over time and new regulatory practices implemented to improve pharmacovigilance processes prior to products becoming available on the market [17]. These improvements have developed alongside evidence-based medicine, influenced by critical incidents, often following product licencing in the post-marketing period, e.g. thalidomide and cerivastatin [18, 19]. Disparities between high-income (HIC) and low- and middle-income country (LMIC) pharmacovigilance processes are well documented, including limited regulatory capacity and lack of resources to undertake pharmacovigilance activities in RCTs or to implement pharmacovigilance regulations [17]. However, LMICs carry the highest disease-burden globally, including neonatal morbidity and mortality, and there is an urgent need for new safe products in clinical practice [20]. Resource constraints and additional barriers to conducting RCTs in the LMIC setting, such as limited pharmacovigilance infrastructure or adaptation of processes to the LMIC setting, need to be overcome to ensure appropriate regulatory oversight and generation of drug safety data where often the need is greatest [17, 21].

The assessment and reporting of AEs in neonates is undoubtedly complex. The presence of multiple co-morbidities in neonates, in particular, those admitted to the neonatal intensive care unit is almost universal. AE grading (intended as a collective term for both AEs and serious adverse events (SAEs)) and classification scales are widely based on adult tools that have been adapted rather than validated for the neonatal setting. There is a lack of comprehensive definitions of expected or unexpected AEs in neonates [3]. Salaets et al. undertook a Delphi consensus process within the International Neonatal Consortium to create a neonatal-specific AE severity scale having identified the lack of a validated neonatal AE severity tool [22]. The resulting Neonatal AE Severity Scale (NAESS) includes 35 common neonatal AEs categorised by organ system. A recent retrospective validation of NAESS performed using RCT data from neonatal trials conducted in the USA showed only moderate reliability, demonstrating the need for enhanced training in the tool prior to use and more detailed AE/safety data collection during neonatal RCTs [22, 23]. Whilst NAESS has been specifically designed for neonates and aims to improve and standardise neonatal AE reporting, it appears to have been primarily developed with high-income settings in mind with limited applicability to the LMIC setting where the burden of common neonatal diseases, particularly neonatal sepsis, is highest. Limitations in intensive care provision (e.g. invasive ventilation, extracorporeal membrane oxygenation) and specialist equipment (e.g. echocardiogram, electroencephalogram), which are often readily available in HICs, mean that assessments of AE severity where there is a reliance on an escalating use of specialised neonatal care, as in NAESS, have limited utility in a low-resource environment.

## Pharmacovigilance in neonatal research and challenges: adaptation to context and next steps

Whilst many challenges exist in the conduct of neonatal trials and the related pharmacovigilance activities and processes, strategies to overcome these are achievable (Table 1).

**Table 1:** Challenges and proposed solutions for safety data collection in neonatal trials

Challenge	Proposed solutions
Limited neonatal-specific randomised controlled trial (RCT) safety data, often due to a reluctance to conduct trials in this population	• Further incentives by regulators and funders to encourage industry partners to conduct RCTs in neonates or to include them in their wider cohorts and thus generate neonatal RCT data • Proportionate safety data collection tailored to the setting, context and intended outputs of the trial, e.g. sponsor-led adaptation of the pharmacovigilance plan within a trial protocol to allow a balanced approach to adverse event reporting, especially for trials where regulatory approval is not being sought. Thus, allowing risk-based reporting, leveraging already known drug safety data to optimise resources whilst maintain participant safety, to encourage RCT data generation in the neonatal population
Lack of globally relevant adverse event assessment tools that take into account disparities in resources and expertise, compromising generalisability of RCTs across different settings	• Development and validation of globally relevant adverse event assessment tools that standardise the classification and severity of AEs across diverse income settings. This should be done in collaboration with research teams and clinicians working within a wide range of settings • Guidance on how to grade AEs based on local context and resource provision can be developed in collaboration with trial clinical teams to facilitate AE reporting and accurate safety reporting in settings where previous trial experience or pharmacovigilance infrastructure may be limited
High burden on trial recruiting sites, which may adversely impact neonates, to collect extensive adverse event data for all neonatal conditions, despite high rates of common co-morbidities, often with short reporting timeframes	• Proportionate safety data collection tailored to the setting, context and intended outputs of the trial, e.g. balanced reporting of safety information relevant to the investigational medicinal product when regulatory approval is not being sought • Use of non-expedited reporting by site investigators for common neonatal conditions not associated with trial drugs • Reporting exemptions for common neonatal conditions not associated with trial drugs, this could also include reporting exemptions for adverse events with a grade of 2 or less • Align clinical data and procedures with routine clinical cares where possible • Avoid extra laboratory tests, where safety risk is low, and when not clinically indicated

When considering the pharmacovigilance approach, including the scope of AE reporting, for any clinical trial, trialists and trial Sponsors should carefully consider the ultimate objective of the trial being conducted. For example, regulatory requirements for the registration of a new product in a neonatal population would require a very different, exhaustive approach to safety data collection compared with a pragmatic, late-phase or public health trial. Collecting AE data in pragmatic, public health trials, where there is a well-established safety profile of the IMP in other populations, should be proportionate and effective and can be tailored to the setting, context and trial population. Avoiding an unnecessarily burdensome one-size fits all approach, given the urgent need to address public health questions, both in epidemic and pandemic settings, requires a clear distinction between a trial that is aiming to identify biological activity of a product (i.e., by measuring efficacy) versus one that aims to establish how drug regimens are used in real-life settings (i.e., measuring effectiveness). Neither of these approaches denies the need to follow regulatory requirements for reporting AEs, but data collection should be risk-based and risk-balanced. The International Conference on Harmonisation (ICH) Good Clinical Practice (GCP) guideline provides extensive guidance on the collection, analysis and reporting of AEs in clinical trials [1]. If it is not an objective of the trial to seek regulatory approval, such as marketing authorisation, sponsors are able, within the remit of GCP, to use discretion to develop an adapted pharmacovigilance strategy where only AEs that are deemed critical to the assessment of safety need to be collected [1]. It is still possible for clinical trials to be conducted with rigour and follow statistically principled methods using this approach. The methodology should be clearly outlined in the protocol and must be informed by the existing evidence available for the safety profiles of the IMPs being investigated and should be considered alongside the variability within the neonatal population and the measures being adopted to assess the severity of each AE. The DOLFIN trial, which investigates developmental outcomes of long-term feed supplementation in neonates, has utilised an adapted approach to SAE reporting where pre-defined and foreseeable SAEs are not reported unless thought to be causally related to IMP [24]. This strategy significantly reduces the burden of recording SAEs for common neonatal conditions in a population known to have high rates of co-morbidities. A strategy to reduce SAE reporting was supported by a systematic review and meta-analysis evaluating safety reporting in 83 paediatric antibiotic trials (21 included neonates), which found paediatric AEs to be predictable and antibiotic class-specific with no unexpected side effects identified [3].

The need for risk-proportionality becomes critically important when clinical trials are conducted in LMIC settings where data collection should be proportionate to the resources and experience of the local teams conducting the trial and have minimal interference with routine clinical care provision. Strengthening capacity in LMIC settings is also key for the conduct of clinical trials in neonates. Strong communication with the wider, non-research, clinical team can enrich the knowledge base around local clinical practices with research learnings underpinning capacity building. Furthermore, high local background mortality rates for conditions such as birth asphyxia, sepsis or pre-term birth should be communicated to institutional review boards (IRB), ethics committees (EC) and competent authorities (CA) to provide regional context to facilitate safety and risk assessment of the trial, particularly in terms of AE expectedness.

Laboratory and clinical assessments to monitor safety within a neonatal RCT should be balanced and aligned with routine clinical care where possible. Laboratory assessments can be a very blunt tool to assess potential AEs in neonates because reference ranges used to define abnormal/out of range results are poorly defined or population-specific, and often do not account for characteristics, such as gestational age. The clinical significance of laboratory abnormalities should also be considered within the context of the neonate’s condition and known comorbidities. Mandating additional blood draws to monitor laboratory parameters to normalisation when not clinically indicated is problematic in such a vulnerable population with small total blood volumes. Monitoring safety events that are expected to resolve following normal physiological processes, such as physiological jaundice, is a good example of this. Furthermore, trial designs can be adapted to pragmatically fit with local clinical care practices and still facilitate trial delivery. For example, meaningful baseline laboratory tests or clinical assessments can be conducted within 24 h of admission, aligning with routine neonatal clinical care so as to minimise unnecessary disruptions. This approach is important as exposure to stressors, such as handling and painful procedures, on the neonatal unit can have a potential impact on neurodevelopmental outcomes [25].

Further work is needed to develop and validate adequate AE assessment tools in neonates, and this should be achieved through a wider consensus amongst experienced investigators, clinicians and triallists with specialist neonatal knowledge; this becomes even more important when working in LMICs where resources are limited making the adoption of current tools restrictive. Clinical-based assessment tools could be considered in low-resource settings where laboratory tests and specialist investigations are not as easily available, and where conditions are often assessed and treated based on clinical signs, and so may better reflect the local clinical practice. Novel methods for optimally assessing safety in this population can be developed based on existing methods, e.g. DAIDS or NAESS [22, 26], adapted and optimised for low resource settings, then evaluated in public health trials. Simplification of current AE assessment tools or the development of tools that can bridge high- as well as low- and middle-income clinical settings would facilitate the global collection of drug safety data in neonates and allow comparison between studies regardless of income setting. This strategy has been adopted in the context of a large, neonatal, public health trial comparing novel combinations of older antibiotics with WHO-recommended regimens as well as other commonly prescribed antibiotic combinations for the empiric treatment of neonatal sepsis in LMICs (NeoSep1, ISRCTN48721236) [27]. The NAESS and neonatal adapted DAIDS adverse event grading tools [22, 26] have been combined to include common neonatal conditions and AE severity grading and then adapted to allow assessment across any neonatal, inpatient setting regardless of the resources or level of care available (Supplementary Material 1). Participating sites completed an equipment and level of care questionnaire which aided in understanding available laboratory tests and medications and not only what and how much equipment was available to support the care of unwell neonates but also the accessibility of expertise to operate this. This allowed a comprehensive understanding of the differences in levels of care across the included trial settings. This has led to an AE grading tool adaptation where AE severity grades can be assigned on the principle of the identified need for escalation of care, whether available or not, including a clinical assessment of the urgency of such an escalation and the threat to life posed by the AE. This approach has led to an AE assessment tool that is highly relevant in any neonatal care setting. This tool along with a companion document (Supplementary Material 2) was developed in full collaboration with participating trial sites and clinicians to guide local research teams on how to grade AEs in their setting regardless of the availability of specialist equipment, medications or investigations at their hospital. However, irrespective of the tool used, it is imperative to ensure research staff are trained to identify, collect and report AE data in a timely and accurate manner. From a legal standpoint, sponsors should design the AE data collection processes and ensure the timely collection and reporting of events is implemented successfully.

## Conclusion

Over the past decade, significant progress has been made by the global academic community and regulators in addressing the gaps in neonatal clinical trials. However, greater effort is required to harmonise research methods around the design and conduct of clinical trials in this important population, including pharmacovigilance processes, to ensure best practices and produce data that are both robust and generalisable. This can be facilitated by ensuring adverse event grading tools are globally relevant and that safety reporting is aligned to the specific trial context, setting and intended outcomes, ultimately advancing the quality and impact of neonatal clinical research.

## Supplementary Information

Supplementary Material 1: Globally Relevant Neonatal Adverse Event Grading Tool. Example of a draft Companion Document for the Globally Relevant Neonatal.

## Data Availability

N/A.

## Abbreviations

AE: Adverse event
CTU: Clinical Trials Unit
CA: Competent authority
DAIDS: Division of AIDS (DAIDS) Table for Grading the Severity of Adult and Pediatric Adverse Events
EC: Ethics committee
IRB: Institutional review board
LMIC: Low- and middle-income country
NAESS: Neonatal Adverse Events Scale
RCT: Randomised controlled trial
SAE: Serious adverse event
WHO: World Health Organization
