# Supplementary material for: Safety reporting in neonatal clinical trials: reflections towards optimal, globally relevant approaches
Source: Trials. 2025 Feb 9;26:46. doi: 10.1186/s13063-025-08723-y (PMC11808976; doi:10.1186/s13063-025-08723-y)
Supplement: Supplementary file 1 — Supplementary Material 1: Globally Relevant Neonatal Adverse Event Grading Tool. Example of a draft Companion Document for the Globally Relevant Neonatal. [file 13063_2025_8723_MOESM1_ESM.pdf]

Supplementary Material 1: Globally Relevant Neonatal Adverse Event Grading Tool

Adapted from the NAESS and neonatal DAIDS adverse event grading tool
(Salaets T, *et al*. Arch Dis Child. 2019; 104:1167-73; IMPAACT (https://www.impaactnetwork.org/studies/p1106))

GLOBALLY RELEVANT NEONATAL ADVERSE EVENT GRADING TOOL

Purpose of this document: to standardise the classification and severity of adverse events across diverse income settings.

In general, and where appropriate, the parameters refer to the associated neonatal conditions e.g., "apnoea" is "neonatal apnoea"; "Respiratory Distress Syndrome" is "Neonatal Respiratory Distress Syndrome", etc.

The requirement for an intervention does not mean that the intervention has to be available e.g., requiring urgent blood transfusion would be the need for an urgent blood transfusion whether the blood was available or not.

Parameter	Grade 0<br>Normal	Grade 1<br>Mild	Grade 2<br>Moderate	Grade 3<br>Severe	Grade 4<br>Potentially Life-Threatening	Grade 5
**RESPIRATORY**						
Apnoea	No apnoeas	Self-limiting apnoea	Apnoea responsive to stimulation<br>**AND/OR**<br>temporary FiO_2 increase	Apnoea requiring sustained FiO_2 increase<br>**AND/OR**<br>requiring non-invasive ventilation<br>**AND/OR**<br>other major care changes required	Apnoea with life-threatening respiratory **and/or** haemodynamic compromise<br>**AND/OR**<br>urgent care change required	Death
Bronchopulmonary Dysplasia	No evidence of BPD by definition	Supplemental oxygen at 28 days<br>**AND**<br>breathing room air<br><br>at 36 weeks postmenstrual age (PMA) in infants born at <32 weeks' gestation<br>**OR**<br>by 56 days postnatal age (PNA) in infants born at >32 weeks gestation<br>**OR**<br>breathing room air at discharge	Supplemental oxygen at 28 days<br>**AND**<br>need for up to 30% oxygen<br><br>at 36 weeks PMA in infants born at <32 weeks' gestation<br>**OR**<br>by 56 days PNA in infants born at >32 weeks gestation<br>**OR**<br>need for up to 30% oxygen at discharge	Supplemental oxygen at 28 days<br>**AND**<br>need for >30% oxygen **OR** positive pressure ventilation<br><br>at 36 weeks PMA in infants born at <32 weeks' gestation<br>**OR**<br>by 56 days PNA in infants born at >32 weeks gestation<br>**OR**<br>need for >30% oxygen **OR** positive pressure at discharge	Supplemental oxygen at 28 days<br>**AND**<br>need for >30% oxygen **AND** positive pressure ventilation<br><br>at 36 weeks PMA in infants born at <32 weeks' gestation<br>**OR**<br>by 56 days PNA in infants born at >32 weeks gestation<br>**OR**<br>need for >30% oxygen **AND** positive pressure at discharge	Death
For conversion of oxygen administered by different modalities to FiO2: see for example http://nicutools.org/MediCalcs/ActualO2.php3						

Parameter	Grade 0<br>Normal	Grade 1<br>Mild	Grade 2<br>Moderate	Grade 3<br>Severe	Grade 4<br>Potentially Life-Threatening	Grade 5
Persistent Pulmonary Hypertension of the Newborn (PPHN)	No signs of PPHN	Evidence of PPHN with no clinical symptoms	Evidence of PPHN with moderate clinical symptoms<br>**AND/OR**<br>an oxygenation index <25<br>**AND/OR**<br>minor care changes required	Evidence of PPHN with severe clinical symptoms<br>**AND/OR**<br>an oxygenation index ≥25 and <40<br>**AND/OR**<br>major care changes required	Evidence of PPHN with life threatening respiratory **and/or** hemodynamic compromise<br>**AND/OR**<br>oxygenation index >40<br>**AND/OR**<br>ECMO required	Death
Pneumothorax	No evidence of pneumothorax	Evidence of pneumothorax with no clinical signs<br>**AND**<br>no care change required	Evidence of pneumothorax with minor clinical signs<br>**AND/OR**<br>minor care changes required	Evidence of pneumothorax with significant clinical signs<br>**AND/OR**<br>major care change	Evidence of pneumothorax with life-threatening respiratory **and/or** haemodynamic compromise<br>**AND/OR**<br>urgent major care change required	Death
Pulmonary Haemorrhage	No evidence of pulmonary haemorrhage	Evidence of pulmonary haemorrhage with no care change required	Evidence of pulmonary haemorrhage without relevant increase in pCO_2 **or** decrease in oxygenation<br>**AND/OR**<br>minor care changes required	Evidence of pulmonary haemorrhage with relevant increase in pCO_2 **or** decrease in oxygenation<br>**AND/OR**<br>major care change required	Evidence of pulmonary haemorrhage with life-threatening respiratory **and/or** hemodynamic compromise	Death
Respiratory Distress Syndrome (RDS)/Insufficiency	No evidence of respiratory distress	Clinical evidence of mildly increased respiratory distress with no apparent change in baseline functioning<br>**AND**<br>no care change required	Clinical evidence of increased respiratory distress with minor care changes required	Clinical evidence of increased respiratory distress with relevant deterioration in gas exchange<br>**AND/OR**<br>major care changes required	Clinical evidence of increased respiratory distress with life-threatening respiratory **and/or** haemodynamic compromise<br>**AND/OR**<br>urgent care changes required	Death
**CARDIOVASCULAR**						
Coagulation disorder	No coagulation abnormality	Minor biochemical coagulation abnormalities without clinical signs<br>**AND**<br>no care change required	Biochemical **or** clinical coagulation abnormalities with clinical signs<br>**AND/OR**<br>increased monitoring required	Biochemical **or** clinical coagulation abnormalities<br>**AND**<br>intervention required	Biochemical **or** clinical coagulation abnormalities with life threatening consequences<br>**AND/OR**<br>urgent major care changes required	Death
Congenital Heart Disease	No congenital heart disease	Minor congenital heart disease<br>**AND**<br>no treatment required	Minor congenital heart disease<br>**AND**<br>future treatment may be required	Major congenital heart disease<br>**AND**<br>no immediate treatment required	Major congenital heart disease<br>**AND**<br>immediate treatment required	Death
Hypertension	No blood pressure (BP) performed	Self-limiting hypertension<br>**AND**<br>no care change required	Persistent hypertension<br>**AND**<br>no care change required	Persistent hypertension<br>**AND**<br>need for antihypertensive medication	Persistent hypertension with life-threatening consequences	Death

Parameter	Grade 0<br>Normal	Grade 1<br>Mild	Grade 2<br>Moderate	Grade 3<br>Severe	Grade 4<br>Potentially Life-Threatening	Grade 5
Hypotension	No BP performed	Hypotension with<br>no effect on perfusion<br>**AND**<br>no care change required	Persistent hypotension with<br>no effect on perfusion<br>**AND/OR**<br>minor care changes required	Persistent hypotension affecting<br>perfusion<br>**AND/OR**<br>major care changes required	Hypotension<br>with life-threatening consequences<br>**AND/OR**<br>urgent care changes required	Death
Oedema	No oedema	Mild oedema<br>**AND**<br>no care change required	Moderate oedema<br>**AND/OR**<br>minor care changes required	Severe oedema<br>**AND/OR**<br>major care change required	Severe oedema<br>with life threatening consequences<br>**AND/OR**<br>urgent major care changes required	Death
Patent Ductus Arteriosus (PDA)	No evidence of PDA	Diagnosis of PDA<br>**AND**<br>no care change required	PDA<br>**AND**<br>minor care changes required	Diagnosis of PDA<br>**AND**<br>major care changes required	Diagnosis of PDA<br>**AND**<br>surgical ligation required	Death
Tachycardia	No tachycardia	Self-limiting episodes of<br>tachycardia<br>**AND**<br>asymptomatic<br>**AND**<br>no care change required	Persistent tachycardia<br>**AND**<br>minor care changes required	Persistent tachycardia resulting in<br>non-life-threatening haemodynamic<br>compromise<br>**AND/OR**<br>major care changes required	Persistent tachycardia resulting in<br>life-threatening consequences<br>**AND/OR**<br>urgent major care changes required	Death
Bradycardia	No bradycardia	Self-limiting episodes of<br>bradycardia<br>**AND**<br>no care change required	Persistent bradycardia<br>**AND**<br>minor care changes required	Persistent bradycardia resulting in<br>non-life-threatening haemodynamic<br>compromise<br>**AND/OR**<br>major care changes required	Persistent bradycardia resulting in<br>life-threatening consequences<br>**AND/OR**<br>urgent major care changes required	Death
**GASTROINTESTINAL**						
Feeding Intolerance	No feeding intolerance	Mild feeding intolerance<br>**AND**<br>no apparent discomfort<br>**AND**<br>no care change required	Moderate feeding intolerance<br>with apparent minor discomfort<br>**or** alteration of drinking<br>behaviour<br>**AND/OR**<br>minor care changes required	Severe feeding intolerance<br>**AND/OR**<br>major change in feeding support<br>required	Severe feeding intolerance<br>**AND**<br>life-threatening consequences	Death
Necrotising Enterocolitis (NEC)	No gastrointestinal dysfunction	*See note*†	*See note*†	NEC confirmed<br>**AND**<br>major care change required	NEC with<br>bowel perforation<br>**AND/OR**<br>life-threatening consequences<br>**AND/OR**<br>urgent major care change required	Death
†If NEC is not confirmed (Bell stages I): please record severity of individual symptoms (e.g. feeding intolerance)						

Parameter	Grade 0<br>Normal	Grade 1<br>Mild	Grade 2<br>Moderate	Grade 3<br>Severe	Grade 4<br>Potentially Life-Threatening	Grade 5
Spontaneous Intestinal Perforation	No evidence of intestinal perforation	-------------------	-------------------	Presence of spontaneous intestinal perforation<br>**AND**<br>non-urgent medical stabilisation and surgical intervention indicated	Presence of spontaneous intestinal perforation resulting in life-threatening consequences<br>**AND**<br>urgent intervention indicated	Death
Vomiting (infantile)	No increase in vomiting from baseline	Increase in vomiting over baseline<br>**AND**<br>self-limiting	Persistent increase in vomiting over baseline with no dehydration<br>**AND/OR**<br>minor changes in feeding support required	Persistent increase in vomiting over baseline with signs of dehydration<br>**AND/OR**<br>major changes in feeding support required	Persistent increase in vomiting with life-threatening consequences	Death
Neonatal Diarrhoea	No diarrhoea	Increase of 2 - 4 stools per day over baseline<br>**OR**<br>mild increase in ostomy output compared to baseline	Increase of 4 - 6 stools per day over baseline<br>**OR**<br>moderate increase in ostomy output compared to baseline	Increase of ≥7 stools per day over baseline<br>**OR**<br>severe increase in ostomy output compared to baseline<br>**AND/OR**<br>signs of dehydration	Neonatal diarrhoea with life-threatening consequences	Death
Neonatal Gastrointestinal (GI) bleeding	No GI bleeding	Mild, self-limiting bleeding<br>**AND**<br>no care change required	Moderate bleeding<br>**AND/OR**<br>minor care change required<br>**AND/OR**<br>change in monitoring required	Severe bleeding<br>**AND/OR**<br>non-life-threatening haemodynamic consequences<br>**AND/OR**<br>major care change required	GI bleeding with life-threatening consequences<br>**AND/OR**<br>urgent major care change required	Death
**HEPATOBILIARY**						
Jaundice	No jaundice	Mild jaundice<br>**AND**<br>no treatment **or** care change required	Jaundice requiring minor care change **AND/OR**<br>change in monitoring	Jaundice requiring major care change	New onset acute bilirubin encephalopathy	Death
**CENTRAL NERVOUS SYSTEM**						
Intraventricular Haemorrhage	No intraventricular haemorrhage noted or not assessed	Germinal matrix haemorrhage	Blood in ventricle<br>**AND**<br>no ventricular enlargement	Blood in ventricle<br>**AND**<br>ventricular enlargement	Parenchymal haemorrhage<br>**AND/OR**<br>ventricular drainage required	Death
Encephalopathy including Hypoxic Ischaemic Encephalopathy	No encephalopathy	-------------------	Mild, transient clinical signs (as per modified Sarnat) of encephalopathy **AND**<br>increased observations required<br>**AND/OR**<br>additional care required	Moderate clinical signs of encephalopathy<br>**AND/OR**<br>meeting the criteria for therapeutic hypothermia	Severe clinical signs of encephalopathy with life threatening consequences	Death

Parameter	Grade 0<br>Normal	Grade 1<br>Mild	Grade 2<br>Moderate	Grade 3<br>Severe	Grade 4<br>Potentially Life-Threatening	Grade 5
Neonatal Convulsion	No convulsions	Single, self-limited suspected seizure<br>**AND**<br>no treatment required	Suspected seizures controlled with 1 anti-seizure drug	Suspected seizures uncontrolled with 1 anti-seizure drug<br>**OR**<br>requiring 2 or more anti-seizure drugs	Suspected seizures with life threatening consequences<br>**AND/OR**<br>suspected status epilepticus‡ despite multiple anti-seizure drugs	Death related to suspected seizures
‡>30 minutes duration of convulsions within a 60-minute period						
Periventricular leukomalacia (PVL)	No PVL<br>**OR**<br>not assessed	Transient periventricular echo densities persisting for > 7 days and resolving completely	Transient periventricular echo densities evolving into small localised frontoparietal cysts or persistent diffuse echodensities	Periventricular echodensities, evolving into extensive cystic periventricular lesions<br>**OR**<br>densities extending into the deep white matter	-------------------	-------------------
Infant Irritability	No irritability	Mild, self-limiting irritability not affecting feeding and sleeping	Moderate irritability<br>**AND**<br>minor changes in feeding and sleeping behaviour<br>**AND/OR**<br>minor additional care required	Severe irritability with major changes in feeding behaviour **and/or** requiring support other than oral feeding<br>**AND/OR**<br>requiring long term medical treatment	Life threatening irritability with loss of autonomic control of temperature **or** heart rate<br>**AND/OR**<br>urgent care changes required	-------------------
Retinopathy of Prematurity (ROP)	Normal vascularisation or not assessed	Incomplete vascularisation<br>**AND**<br>no care changes required	Pre-threshold ROP<br>**AND/OR**<br>more frequent ophthalmic monitoring than routine	Threshold ROP<br>**AND/OR**<br>major care changes required	Unilateral retinal detachment	Blindness (bilateral retinal detachment, …..)
**GENITOURINARY/RENAL**						
Renal Dysfunction	Wet nappies/diapers documented	Evidence of mild renal dysfunction	Evidence of moderate renal dysfunction	Evidence of severe renal dysfunction	Evidence of life-threatening renal dysfunction	Death
**INFECTIONS/INFESTATIONS**						
Sepsis<br>(Culture positive or Culture negative)	No signs or symptoms of sepsis	Evaluation for sepsis<br>**AND**<br>no anti-infectives started	Suspected sepsis with mild or ambiguous signs<br>**AND/OR**<br>anti-infectives initiated	Sepsis with severe signs<br>**AND/OR**<br>supportive care initiated **or** escalated<br>**AND/OR**<br>anti-infective treatment escalated<br>**AND**<br>no signs of septic shock **and/or** meningitis	Sepsis with life-threatening consequences<br>**AND**<br>urgent major care change required	Death

Parameter	Grade 0<br>Normal	Grade 1<br>Mild	Grade 2<br>Moderate	Grade 3<br>Severe	Grade 4<br>Potentially Life-Threatening	Grade 5
**BLOOD/ELECTROLYTE/METABOLIC DISORDERS/LABORATORY ABNORMALITIES**<br>Only to be reported if deemed clinically significant						
Anaemia	No anaemia	Anaemia<br>**AND**<br>according to clinical judgment no additional monitoring required	Anaemia with more frequent monitoring required	Anaemia with no clinical signs<br>**AND**<br>requires blood transfusion	Anaemia with clinical signs of shock<br>**AND**<br>requires blood transfusion	Death
Electrolyte/Metabolic Disorders	No electrolyte/metabolic disorder	Electrolyte/metabolic disorder<br>**AND**<br>according to clinical judgment no treatment or additional monitoring required	Electrolyte/metabolic disorder with no systemic signs<br>**AND**<br>more frequent monitoring required<br>**AND/OR**<br>minor care changes required	Electrolyte/metabolic disorder requiring intravenous correction	Electrolyte/metabolic disorder with systemic signs<br>**AND/OR**<br>urgent invasive treatment required	Death
Leukopaenia	No leukopaenia	Leukopaenia<br>**AND**<br>according to clinical judgment no additional monitoring required	Leukopaenia with more frequent monitoring required	Clinically relevant leukopaenia requiring treatment<br>**AND/OR**<br>major care changes required	Life threatening leukopaenia<br>**AND/OR**<br>urgent major care change required	Death
Neutropaenia	No neutropaenia	Neutropaenia<br>**AND**<br>according to clinical judgment no additional monitoring required	Neutropaenia with more frequent monitoring required	Clinically relevant neutropaenia<br>**AND/OR**<br>GCSF treatment required<br>**AND/OR**<br>major care changes required	Life-threatening neutropaenia<br>**AND/OR**<br>white cell transfusion required<br>**AND/OR**<br>urgent major care changes required	Death
Thrombocytopenia	No thrombocytopaenia	Thrombocytopaenia<br>**AND**<br>according to clinical judgment no additional monitoring required	Thrombocytopaenia with more frequent monitoring required	Thrombocytopaenia with non-life-threatening bleeding	Life-threatening thrombocytopaenia with associated life-threatening bleeding<br>**AND/OR**<br>platelet transfusion required<br>**AND/OR**<br>urgent major care changes required	Death
**OTHER**						
Administration site complication	No administration site complication	Painless oedema	Erythema with associated symptoms	Ulceration<br>**AND/OR**<br>necrosis<br>**AND/OR**<br>severe tissue damage<br>**AND/OR**<br>operative intervention indicated	Life-threatening consequences<br>**AND/OR**<br>urgent intervention required	Death

Parameter	Grade 0<br>Normal	Grade 1<br>Mild	Grade 2<br>Moderate	Grade 3<br>Severe	Grade 4<br>Potentially Life-Threatening	Grade 5
Neonatal rash	No rash	Localised rash	Diffuse rash<br>**AND/OR**<br>target lesions	Diffuse rash **or** vesicles<br>**AND/OR**<br>limited number of bullae<br>**AND/OR**<br>superficial ulcerations of mucous membrane limited to one site	Extensive **or** generalised bullous lesions<br>**AND/OR**<br>ulceration of mucous membranes involving ≥2 distinct mucosal sites<br>**AND/OR**<br>Stevens Johnson syndrome<br>**AND/OR**<br>toxic epidermal necrosis	Death
Congenital Anomalies	No congenital abnormalities	Minor congenital abnormality with no impairment of function	Minor congenital abnormality with no impairment of function<br>**AND**<br>future treatment may be needed	Major congenital abnormality with impairment of function<br>**AND**<br>no immediate treatment needed but future treatment may be needed	Major congenital abnormality with impairment of function<br>**AND**<br>urgent treatment needed	Death
Neonatal Abstinence Syndrome (NAS)	No history of NAS	NAS with signs<br>**AND**<br>no medical treatment required	NAS controlled with a single drug	NAS controlled with two drugs	NAS with seizures	Death
Any other AE	-------------------	Mild presentation<br>**AND**<br>asymptomatic **or** mild symptoms<br>**AND**<br>clinical **or** diagnostic observations only<br>**AND**<br>no change in baseline age-appropriate behaviour*<br>**AND**<br>no change in baseline care **or** monitoring indicated	Moderate presentation resulting in minor changes of baseline age-appropriate behaviour*<br>**AND/OR**<br>minor changes in baseline care **or** monitoring required***	Severe presentation resulting in major changes of baseline age-appropriate behaviour* **or** non-life-threatening changes in basal physiological processes**<br>**AND/OR**<br>major change in baseline care or monitoring required****	Life-threatening presentation resulting in life threatening changes in basal physiological processes**<br>**AND/OR**<br>urgent major change in baseline care required	Death

*Age-appropriate behaviour refers to oral feeding behaviour, voluntary movements and activity, crying pattern, social interactions and perception of pain. **Basal physiological processes refer to oxygenation, ventilation, tissue perfusion, metabolic stability organ functioning. ***Minor care changes constitute: brief, local, non-invasive or symptomatic treatments. ****Major care changes constitute: surgery, addition of long-term treatment, upscaling care level

BP = blood pressure; GI = gastrointestinal; Hb = haemoglobin; NEC = necrotising enterocolitis; PDA = patent ductus arteriosus; PMA = postmenstrual age; PNA = postnatal age
PPHN = persistent pulmonary hypertension of the newborn; PVL = periventricular leukomalacia; RDS = respiratory distress syndrome; ROP = retinopathy of prematurity

Supplementary Material 2: Example of a draft Companion Document for the Globally Relevant Neonatal Adverse Event Grading Tool

GLOBALLY RELEVANT NEONATAL ADVERSE EVENT GRADING TOOL COMPANION DOCUMENT

DRAFT DOCUMENT

The investigations and interventions provided by trial sites will vary dependent on the level of care they can provide/resources/availability of specialist healthcare equipment and personnel. This document has been designed to be a companion to the Globally Relevant Neonatal Adverse Event Grading Tool adapted, with reference to the NAESS and neonatal DAIDS AE grading tools [1,2], to standardise the classification and severity of adverse events across diverse incomes settings. It can be used to provide further information on how adverse events should be graded. This document outlines some of the ways of diagnosing and managing specific neonatal conditions but it is not expected that all sites will be able to provide all diagnostic or management tools mentioned. Information is only provided where needed and so “Diagnosis” and “Management” sections are not provided for all conditions.

An adverse event (AE) can be graded on the need for a particular treatment/management option whether it is available or not e.g. Patent Ductus Arteriosus should be graded as Grade 3 if the PDA was deemed by the clinician to require treatment with indomethacin or ibuprofen whether these medications are available at their site or not.

In general, and where appropriate, the parameters refer to the associated neonatal conditions e.g., “apnoea” is “neonatal apnoea”; “Respiratory Distress Syndrome” is “Neonatal Respiratory Distress Syndrome”, etc.

Contents

RESPIRATORY DISORDERS 9
CARDIOVASCULAR DISORDERS 11
GASTROINTESTINAL DISORDERS 13
HEPATOBILIARY DISORDERS 14
CENTRAL NERVOUS SYSTEM DISORDERS 15
GENITOURINARY DISORDERS 16
INFECTIONS/INFESTATIONS 16
BLOOD/ELECTROLYTE/METABOLIC DISORDERS/LABORATORY ABNORMALITIES 17
OTHER DISORDERS 18

RESPIRATORY DISORDERS

For conversion of oxygen administered by different modalities to FiO_2: see for example http://nicutools.org/MediCalcs/ActualO2.php3

Apnoea

Diagnosis

Apnoea spell defined as apnoea event >20s or associated with bradycardia, hypoxia or cyanosis

Persistent pulmonary hypertension (PPHN)

Diagnosis

Diagnosis of PPHN might be made solely on clinical examination and medical history. Diagnosis of PPHN might also be made following investigations (when available) and could include:

- increased estimated right ventricular pressure on echocardiography
- pre/post-ductal saturation difference of >10%

Management

Minor care change required (whether available or not) could include: supplemental oxygen, non-invasive ventilation

Major care change required (whether available or not) could include: need for invasive ventilation, inhaled nitric oxide (iNO), sildenafil

ECMO required – this is the need for ECMO whether it is available or not

Pneumothorax

Diagnosis

Evidence of pneumothorax could include: radiological evidence (e.g. a small pneumothorax detected on chest X-ray performed for a different reason).

Pneumothorax might also be diagnosed on clinical examination

Evidence of a pneumothorax with life-threatening consequences could include: tension pneumothorax

Management

Minor care change required (whether available or not) could include: increased monitoring, supplemental oxygen

Major care change required (whether available or not) could include: need for a chest drain

Pulmonary haemorrhage

Diagnosis

Evidence of pulmonary haemorrhage could include: haemorrhagic secretion in the endotracheal tube deemed to be pulmonary in origin

Management

Minor care changes (whether available or not) required could include: increase in positive end expiratory pressure.

Major care change (whether available or not) could include: change in ventilatory support, need for blood transfusion

Respiratory Distress Syndrome/Insufficiency

Diagnosis

Signs of respiratory distress syndrome (RDS) could include increased work of breathing, deterioration in gas exchange e.g. increase in pCO_2 or decrease in oxygenation on blood gas (if available)

Mild disease, i.e. Grade 1, could include: neonate is self-ventilating in air; no significant deterioration in gas exchange (e.g. increase in pCO_2 or decrease in oxygenation on blood gas (if available); radiological evidence of RDS without clinical signs

Moderate disease, i.e. Grade 2, could include: clinical evidence of increased respiratory distress with no significant change to gas exchange e.g. no significant increase in pCO_2 or decrease in oxygenation on blood gas (if performed)

Severe disease, i.e. Grade 3, could include: clinical evidence of increased respiratory distress with relevant deterioration in gas exchange e.g. significant increase in pCO_2 or decrease in oxygenation on blood gas (if performed)

Management

Mild disease, i.e. Grade 1 = No supportive care required; neonate is self-ventilating in air

Minor care change required (whether available or not) could include: minor adjustments in current ventilatory support, new supplemental oxygen requirement or need for non-invasive support (e.g. CPAP/BiPAP/HHFNC)

Major care change required (whether available or not) could include: need for invasive ventilation

Urgent major care change required (whether available or not) could include: urgent intubation

CARDIOVASCULAR DISORDERS

Bradycardia

Diagnosis

Mild disease, i.e. Grade 1, could include brief and/or self-limiting episodes of bradycardia

For moderate, severe and life-threatening disease, i.e. Grades 2 – 4, the bradycardia should be persistent

Life-threatening consequences could include: shock

Management

Minor care changes required could include: change to concomitant medication, increased monitoring, treatment started, intermittent increase in oxygen requirement

Major care changes required (whether available or not) could include: escalation of Grade 2 treatment; need for new medication; need for intervention

Coagulation disorder

Diagnosis

Biochemical **or** clinical coagulation abnormalities with life-threatening consequences could include: severe pulmonary embolism, limb ischaemia, haemorrhagic shock, disseminated intravascular coagulation (DIC)

Management

Intervention for coagulation disorder could include (whether available or not): correction of coagulation; blood transfusion

Urgent care changes could include (whether available or not): urgent blood transfusion

Congenital heart disease

Diagnosis

Minor congenital heart disease = no impairment to function

Major congenital heart disease = impairment to function

Management

Immediate treatment (whether available or not) could include: prostaglandin infusion, urgent intubation

Hypertension

Diagnosis

Persistent hypertension with life-threatening consequences could include: malignant hypertension shock; cardiac failure; neonatal encephalopathy

Management

Need for anti-hypertensive medication is based on the neonate requiring anti-hypertensive medication (whether this is available or not)

Hypotension

Diagnosis

Persistent hypotension with life-threatening consequences could include: shock, organ failure

Management

Minor care change required (whether available or not) could include: additional fluids
Major care change required (whether available or not) could include: need for medication e.g. vasoactive drugs (inotropes) or hydrocortisone

Oedema

Diagnosis

Severe oedema with life-threatening consequences could include: respiratory failure, shock

Management

Minor care changes required could include: alteration in fluid management

Major care change required (whether available or not) could include: need for diuretics

Urgent care change required (whether available or not) could include: intubation, dialysis

Patent ductus arteriosus

Management

Minor care changes required (whether available or not) could include: treatment with fluid restriction; treatment with diuretics

Major care changes required (whether available or not) could include: treatment with indomethacin, ibuprofen, paracetamol

Tachycardia

Diagnosis

Mild disease, i.e. Grade 1, could include brief and/or self-limiting episodes of tachycardia

For moderate, severe and life-threatening disease, i.e. Grades 2 – 4, the tachycardia should be persistent

Life-threatening consequences could include: shock

Management

Minor care changes required could include: change to concomitant medication, increased monitoring

Major care changes required (whether available or not) could include: escalation of Grade 2 treatment; need for new medication; need for intervention

GASTROINTESTINAL DISORDERS

Diarrhoea

Diagnosis

Neonatal diarrhoea with life-threatening consequences could include: severe dehydration

Feeding intolerance

Diagnosis

Mild feeding intolerance could include: increased gastric residual volume, abdominal distension

Severe feeding intolerance with life-threatening consequences could include: severe dehydration

Management

Minor care changes required (whether available or not) could include: feeds withheld

Major care changes required (whether available or not) could include: change to total parenteral nutrition, change to nasogastric/orogastric feeding

Gastrointestinal bleeding

Diagnosis

Gastrointestinal bleeding with life-threatening consequences could include: haemorrhagic shock

Management

Major care changes required (whether available or not) could include: invasive intervention, transfusion, long term medical treatment

Urgent care change required (whether available or not) could include: urgent transfusion, urgent intubation

Necrotising enterocolitis

Diagnosis

Potentially life-threatening NEC, i.e. Grade 4, could include: NEC with shock (hypotension, bradycardia), disseminated intravascular coagulation, neutropaenia, combined respiratory and metabolic acidosis (Bell IIIA), pneumoperitoneum (Bell IIIB)

Management

Major care changes required (whether available or not) could include: nil by mouth, antibiotic treatment, non-urgent surgery

Urgent major care changes required (whether available or not) could include: urgent surgery

Spontaneous intestinal perforation

Spontaneous intestinal perforation with life-threatening consequences could include: shock, organ failure

Vomiting (infantile)

Diagnosis

Persistent increase in vomiting with life-threatening consequences could include: severe dehydration

Management

Major changes in feeding support required may include: change to naso/orogastric feeding when not previously needed, change to TPN when not previously needed

HEPATOBILIARY DISORDERS

Jaundice

Management

Moderate disease, i.e. Grade 2, could include jaundice requiring phototherapy and/or intravenous immunoglobulin, supportive treatment e.g. fat soluble vitamins, ursodeoxycholic acid

Severe disease requiring major care change, i.e. Grade 3, could include jaundice requiring exchange transfusion (whether available or not)

CENTRAL NERVOUS SYSTEM DISORDERS

Encephalopathy

Diagnosis

Meeting criteria for therapeutic hypothermia = Meeting criteria for therapeutic hypothermia whether this is offered or not

Management

Life-threatening consequences could include: respiratory depression, refractory seizures

Intraventricular haemorrhage

Diagnosis

Life-threatening disease, i.e. Grade 4, could include: parenchymal haemorrhage

Management

Management of life-threatening disease, i.e. Grade 4, could include (whether available or not): ventricular drainage

Convulsions

Diagnosis

Suspected seizures with life-threatening consequences could include: respiratory depression, need for ventilation, refractory seizures

Status epilepticus = >30 minute duration of convulsions within any 60-minute period

Irritability

Management

Minor additional care required could include: occasional analgesics

Long-term medical treatment could include: sedatives

Retinopathy of prematurity

Diagnosis

Blindness/bilateral retinal detachment should be graded as Grade 5

Management

Major care changes could include: any treatment for ROP such as laser intervention, intravitreal anti-VEGF, operative management

GENITOURINARY DISORDERS

Renal dysfunction

Diagnosis

For categorisation of renal dysfunction refer to the below Neonatal KDIGO table [3]

- Mild renal dysfunction could include: KDIGO Stage 0 AKI
- Moderate renal dysfunction could include: KDIGO Stage 1 AKI
- Severe renal dysfunction could include: KDIGO Stage 2 AKI
- Life-threatening renal dysfunction could include: KDIGO Stage 3 AKI

Neonatal KDIGO – can be staged on either serum creatinine **AND/OR** urine output

AKI stage	Serum creatinine	Urine output (hourly)	Grading
**0**	No change in SCr **OR** SCr rise <0.3 mg/dL	≥0.5 ml/kg/hour	Mild – Grade 1
**1**	SCr rise of ≥0.3 mg/dL within 48 hours **OR** SCr rise ≥ 1.5 – 1.9 x baseline SCr*	<0.5 ml/kg/hour x 6 – 12hours	Moderate – Grade 2
**2**	SCr rise ≥2.0 – 2.9 x baseline SCr	< 0.5 ml/kg/hour >12 hours	Severe – Grade 3
**3**	SCr rise ≥3 x baseline **OR** ≥2.5 mg/dL** **OR** kidney support therapy utilisation	<0.3 ml/kg/hour for ≥24 hours **OR** anuria for ≥12 hours	Life-threatening – Grade 4

mg/dL – milligrams per decilitre; CR = serum creatinine; *Baseline SCr defined as lowest previous SCr value; **SCr value of 2.5 mg/dL represents glomerular filtration rate of <10 mL/min/1.73 m2

Adapted from Kidney Disease: Improving Global Outcomes (KDIGO Acute Kidney Injury Workgroup. KDIGO clinical practice guideline for acute kidney injury. Kidney Int Suppl 2012;2:1–138

INFECTIONS/INFESTATIONS

Sepsis

Diagnosis

Severe signs of sepsis could include: fever, grunting, **no** signs of septic shock or meningitis

Sepsis with life-threatening consequences could include: shock, disseminated intravascular coagulation

BLOOD/ELECTROLYTE/METABOLIC DISORDERS/LABORATORY ABNORMALITIES

Anaemia

Diagnosis & Management

Mild disease, i.e. Grade 1, is the presence of anaemia and where according to clinical judgement no additional monitoring is deemed necessary. Haemoglobin, if performed, might, in general, be in the range of 8 – 10 g/dL with no need for a blood transfusion and no additional monitoring as per the clinician's judgement

Moderate disease, i.e. Grade 2, is the presence of anaemia but where according to clinical judgement more frequent monitoring is deemed necessary but blood transfusion is not needed. Haemoglobin, if performed, might in general be ≤8 g/dL with additional monitoring but no need for a blood transfusion as per the clinician's judgement

Severe disease, i.e. Grade 3, is the presence of anaemia without any clinical signs that according to clinical judgement requires a blood transfusion, whether available or not

Electrolyte/Metabolic Disorders

Management

Mild disease, i.e. Grade 1, is the presence of an electrolyte/metabolic disorder but where according to clinical judgement no treatment or additional monitoring are deemed necessary.

Moderate disease, i.e. Grade 2, is the presence of an electrolyte/metabolic disorder with no systemic signs and where according to clinical judgement more frequent monitoring or minor care changes are deemed necessary. More frequent monitoring required could include: repeating blood tests for electrolyte levels more often than usual care. Minor care changes could include (whether available or not): oral/nasogastric/orogastric electrolyte correction

Severe disease, i.e. Grade 3, is the presence of an electrolyte/metabolic disorder requiring intravenous electrolyte correction, whether available or not

Leukopaenia

Diagnosis & Management

Mild disease, i.e. Grade 1, is the presence of leukopaenia and where according to clinical judgement no additional monitoring is deemed necessary.

Moderate disease, i.e. Grade 2, is the presence of leukopaenia but where according to clinical judgement more frequent monitoring is deemed necessary

Severe disease, i.e. Grade 3, is the presence of clinically-relevant leukopaenia requiring treatment, whether available or not and/or major care changes are required

Life-threatening leukopaenia, i.e. Grade 4, is the presence of life-threatening leukopaenia requiring urgent major care changes and/or treatment. Life-threatening leukopaenia could include: leukopaenia with secondary life-threatening sepsis

Neutropaenia

Diagnosis & Management

Mild disease, i.e. Grade 1, is the presence of neutropaenia and where according to clinical judgement no additional monitoring is deemed necessary. Absolute neutrophil count (ANC), if performed, might, in general be $<1000/mm^3$

Moderate disease, i.e. Grade 2, is the presence of neutropaenia but where according to clinical judgement more frequent monitoring is deemed necessary. ANC, if performed, might, in general, be $<500/mm^3$

Severe disease, i.e. Grade 3, is clinically relevant neutropaenia where a major care change is required and/or GCSF treatment is deemed necessary, whether available or not

Life-threatening disease, i.e. Grade 4, is the presence of life-threatening neutropaenia requiring urgent major care changes and/or white cell transfusion, whether available or not. Life-threatening neutropaenia could include: neutropaenia with secondary life-threatening sepsis

Thrombocytopaenia

Diagnosis & Management

Mild disease, i.e. Grade 1, is the presence of thrombocytopaenia and where according to clinical judgement no additional monitoring is deemed necessary. Platelet count, if performed, might, in general, be in the range of 75 – 100 $x10^9/L$ with no need for a platelet transfusion and no additional monitoring as per the clinician's judgement

Moderate disease, i.e. Grade 2, is the presence of thrombocytopaenia but where according to clinical judgement more frequent monitoring is deemed necessary but platelet transfusion is not needed. Platelet count, if performed, might, in general, be in the range of 50 < 75 $x10^9/L$ with additional monitoring but no need for a platelet transfusion as per the clinician's judgement

Severe disease, i.e. Grade 3, is the presence of thrombocytopaenia with non-life threatening bleeding that according to clinical judgement does not require a platelet transfusion. Platelet count, if performed, might, in general, be in the range of 25 < 50 $x10^9/L$ without life-threatening bleeding.

Life-threatening disease, i.e. Grade 4, is the presence of life-threatening thrombocytopaenia with associated life-threatening bleeding and/or where according to clinical judgement a platelet transfusion is deemed necessary and/or major care changes are required. Platelet count, if performed, might in general, be <25 $x10^9/L$

OTHER DISORDERS

Administration site complication

Associated symptoms could include: oedema, pain, induration, phlebitis

REFERENCES

1. IMPAACT: Pharmacokinetic characteristics of antiretrovirals and tuberculosis medicine in low birth weight infants protocol [Internet]. 2018 [cited 2024 Oct 13]. Available from: https://www.impaactnetwork.org/studies/p1106

2. Salaets T, Turner MA, Short M, Ward RM, Hokuto I, Ariagno RL, et al. Development of a neonatal adverse event severity scale through a Delphi consensus approach. Arch Dis Child. 2019;104:1167–73.

3. Selewski DT, Charlton JR, Jetton JG, Guillet R, Mhanna MJ, Askenazi DJ, et al. Neonatal Acute Kidney Injury. Pediatrics. 2015;136:e463–73.
